# Status of Prevention of Mother-to-Child Transmission (PMTCT) Services Utilization and Factors Affecting PMTCT Service Uptake by Pregnant Women Attending Antenatal Care Clinic in Selected Health Facilities of Afar Regional State, Ethiopia

**DOI:** 10.1155/2018/5127090

**Published:** 2018-12-12

**Authors:** Chalachew Genet Akal, Dessie Tegegne Afework

**Affiliations:** ^1^MSc in Medical Microbiology, College of Medicine and Health Science, Bahir Dar University, Bahir Dar, Ethiopia; ^2^MSc in Medical Microbiology, Department of Medical Laboratory Science, Debre Tabor University, Debre Tabor, Ethiopia

## Abstract

Mother-to-child transmission (MTCT) is the predominant way for children to acquire human immunodeficiency virus (HIV) infection worldwide including Ethiopia. Thus, objective of this study was to determine the status of prevention of mother-to-child transmission (PMTCT) services utilization and factors affecting PMTCT utilization in health facilities of Afar region, Ethiopia. A cross-sectional study was conducted from December 2014 to April 2015 taking 347 pregnant women and 22 health care providers. Data were collected using a questioner prepared separately for pregnant women and health care providers involved in PMTCT service delivery. Data were analyzed using SPSS considering *P* value <0.05 statistical significant. The study indicated that the PMTCT service utilization was 67.7%. The study also showed that there is statistically significant association in using PMTCT service with women education level, monthly income, and residence around PMTCT site. Though not statistically significant, excess waiting time, limited physical access to PMTCT sites, and transportation problem were identified as barriers for PMTCT service utilization by pregnant women. Though knowledge of mothers on MTCT of HIV and PMTCT service utilization in agropostural community of Afar region was promising, there were also different barriers identified hindering PMTCT utilization. Thus, we recommend “Wored” and zonal health office to create awareness on significance of PMTCT service in the community, enhance accessibility of PMTCT sites, provide up-to-date trainings for health care providers, and ensure constant supply for PMTCT service.

## 1. Introduction

Women living with human immunodeficiency virus (HIV) infection can give birth to infants infected with HIV which is known as mother-to-child transmission (MTCT) contributing major proportion for new HIV infections among children. Mother-to-child transmission of HIV is causing significant impact contributing 700,000 estimated new HIV infections in children annually in the globally [1]. In 2016, there were 1.8 million children living with HIV globally where 90% of the infections were acquired through MTCT of HIV [2]. Based on 2016 World Health Organization (WHO) data, around 76% of all pregnant women living with HIV globally received medicines that prevent HIV transmission to their babies [3]. WHO recommends countries, including Ethiopia, to implement prevention of mother-to-child transmission (PMTCT) as it is the most effective strategy for preventing HIV to the pediatrics [4]. In Ethiopia, even though the number of sites and PMTCT coverage vary among region, the average PMTCT service coverage was 54.9% in 2012/13. Moreover, the national coverage of antiretroviral therapy for HIV-positive pregnant mothers in 2012 was 42.9%, and the Afar regional state coverage (the present study site) was only 20% in the same year [5]. Without preventive interventions, more than a third of the infants born to HIV-positive mothers will contract HIV [6].

Different countries, including Ethiopia, are providing PMTCT services in different health facilities as part of antenatal care (ANC). Despite an increase in the number of health facilities (HFs) providing PMTCT services in Ethiopia, the proportion of HIV-positive pregnant women who receive antiretroviral (ARV) drugs for PMTCT remains low. In 2009, only 8 percent of HIV-positive pregnant women received antiretroviral prophylaxis. As a result, many women end up grappling with their HIV status unaided and alone [5, 7]. Moreover, Ethiopia is implementing universal HIV screening of pregnant women since 2007 and working to achieve 90-90-90 treatment target endorsed in the 2016 United Nations political declaration on ending AIDS. Moreover, Ethiopia is also working to eliminate MTCT of HIV by 2020 [2].

As being reported by different studies, different factors influence utilization of PMTCT services directly or indirectly: physical accessibility, inconsistent supply of resources for the service, testing methods, lack of trust on result confidentiality, fear of stigma and discrimination, treatment for mothers, feasibility of replacement feeding, inadequate counseling rooms, extent of male involvement in the decision of HIV testing and ANC clinics follow up, lack of information about the presence of the service, economical status, educational level of women, lack of awareness on the significance of voluntary counseling and testing (VCT) and PMTCT, shortage of qualified health professionals, lack of up-to-date training for health care providers, high health care provider turnover, and insufficient allocation of budget [8–10].

Identifying utilization of PMTCT and barriers affecting the utilization of PMTCT service will allow to identify the gap and act accordingly so as to make the service accessible to all pregnant women who need PMTCT service. But up to our knowledge, there is no study done to assess utilization of PMTCT service and associated factors acting as a barrier for the service. Thus the objective of the present study is to determine utilization of PMTCT services and associated factors among pregnant women attending ANC clinic in selected health facilities of Afar region, Ethiopia.

## 2. Methods and Materials

A cross-sectional study was conducted from December 2014 to April 2015 in the Afar regional state of Ethiopia. The Afar regional state is one of the 9 national regional states and two city administrations comprising the Federal Democratic Republic of Ethiopia. The region has a total population of 1,411,092 accounting 1.7% of the total Ethiopian population [11]. The region has 3 hospitals, 23 health centers, and 238 health posts as well as 7 health facilities providing PMTCT service as part of ANC services at the time of study. The present study was conducted in randomly selected 5 governmental health facilities (Dubti Hospital, Aysaita District Hospital, Samara Health center, Logia health center, and Chifra Health Center) providing PMTCT service based on provider-initiated HIV testing and counseling approaches.

From the five health facilities, 347 pregnant mothers attending ANC service were included in the study which was determined using a single proportion formula and by considering design effect of 2 and 10% nonresponse rate assumption. In addition to 347 pregnant women attending ANC service, all 22 health professionals in 5 selected health facilities providing PMTCT service as part of ANC were included in the study to identify factors affecting PMTCT service utilization. The 347 study participants (pregnant women) were proportionally allocated for 5 health facilities providing PMTCT service based on the total population they serve (i.e., the highest study participants were recruited from the health facility serving the highest population). After proportional allocation, convenient sampling technique was used for each health facility until the required number is reached. Then, the data were collected both from selected pregnant women visiting ANC and health professionals using the pretested questioner which was developed in English and translated to the local language (Amharic). Before data collection started, the “Amharic” questioner was back translated into English for validation. Ten trained data collectors who have diploma in health science were involved in data collection through face-to-face interview supervised by three supervisors after obtaining ethical clearance from Samara University and permission letter from Afar Regional Health Bureau. The questioner was designed to collect data on sociodemographic, acceptability of PMTCT service by local community, and associated factors in the utilization of PMTCT service.

To maintain data quality, the questioner was pretested in Awash Arba health center which was not part of the present study. Moreover, every questioner was checked by data collectors and supervisors for inconsistency and incompleteness at the end of data collection. The collected data were analyzed using SPSS for different descriptive statistics, and associations were assessed by using the *x*^2^ test. The bivariant logistic regression analyses were used to see the relation between dependent variable and independent variables considering a *P* value <0.05 as statistically significant. Finally, the adjusted odds ratio was used to see the strongest predictors and avoid the confounders.

## 3. Results

From 347 pregnant women included in the study, 34.9% were 19–25 years old and 57.1% of were from hospitals. Moreover, 72.9% and 54.6% of pregnant women included were from urban and have monthly income less than 1000 Ethiopian birr (ETB), respectively. Furthermore, the study also showed that 89.3%, 77.2%, 35.7%, and 50.1% pregnant women participated in the study were married, Muslims, illiterate, and housewives, respectively (Table 1).

**Figure 1:**
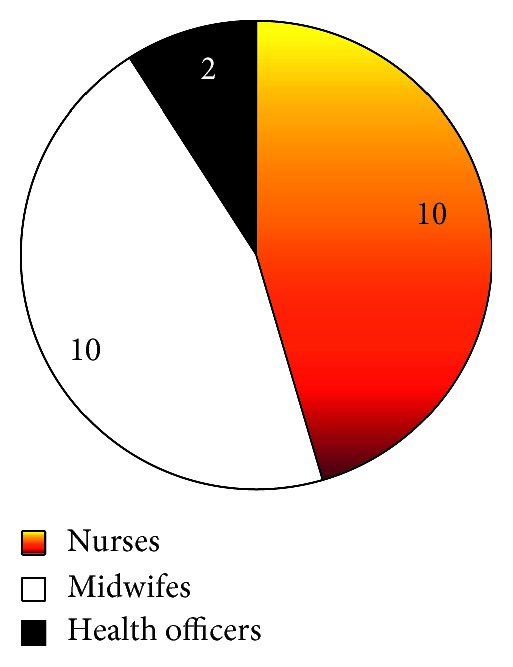
Professional mix of health professionals providing PMTCT service in five health facilities studied from December 2014 to April 2015 in Afar regional state, Ethiopia.

The study indicated that 22 health professionals were providing PMTCT services (Figure 1) with a mean age of 26.4 years. Among 22 health professionals, 16 of them were females. The health professionals in each three health facilities (Chifra Health Center, Logia health center, and Aysaita hospital) were 4. The rest Dupti hospital and Samara Health center have 7 and 3 health professionals, respectively. In the study, 16 of the PMTCT providers had taken PMTCT training at least once in their life time.

Time of ANC follow up initiation varied among pregnant women from 14 to 16 weeks by 59.7% to 24–28 weeks by 17%, respectively. The decision to start ANC service was made by themselves, by husband/partner involvement, and health professional involvement in 57.6%, 36.3%, and 6.1%, respectively. Being asked about why they attended ANC service, 32.46%, 22.2%, 18.7%, 6.3%, and 4.9% of pregnant women replied that they attended ANC to know the status of their pregnancy and their fetus health, to get vaccine, to get treatment for their disease, and to get family planning service after delivery as well as to test for HIV so as to use PMTCT service, respectively. The study also showed that 46.4% of the study participants have knowledge about MTCT of HIV.

**Figure 2:**
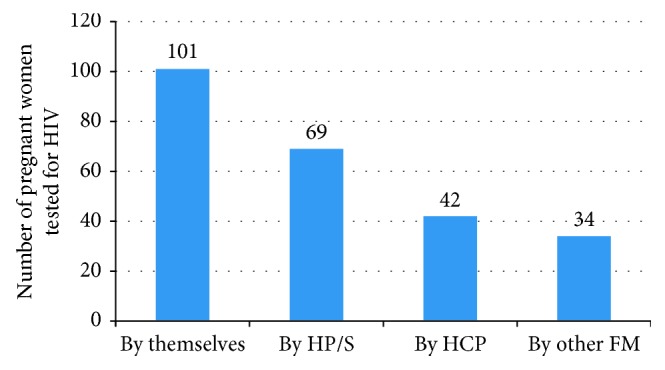
The involvement of different parties in the decision of pregnant women for HIV testing during their ANC follow up in selected health facilities of Afar region, Ethiopia from December 2014 to April 2015. HP/S: husband or partner support; HCP: health care provider; FM: family members.

In the study, 246 (70.9%) pregnant women were tested for HIV where 81.3%, 13.8%, and 4.9% of them were negative, positive, and did not know their status, respectively. Among HIV-positive pregnant women, 23 of them took ART drugs and prophylaxis for their babies making utilization of PMTCT service in the study area 67.7% (Figure 2). Moreover, pregnant women asked on their baby feeding habit/plan, and all preferred to feed breast milk than formula feeding irrespective of their HIV status.

Being asked about their reason why they were not voluntary to be tested for HIV during their ANC follow up, 34 (33.7%) of them reported for the lack of awareness and knowledge followed by doubt on result confidentiality (24.7%), absence and carelessness of health professionals providing PMTCT service (21.8%), lack of interest (9.8%), tested before (7%), and fear of stigma (3%). Most of the pregnant women (46.4%) attending ANC follow up believed that HIV can be transmitted from mother to their child. But, 29.4% believed that it cannot be transmitted and 24.2% did not know any information. Among pregnant women who believed HIV transmission from mother to new born, 33.5% reported that the transmission is in three ways (during pregnancy, during delivery, and through breast feeding), while 31.1%, 24.2%, and 11.2% believed only through delivery, during pregnancy, and through breast feeding, respectively.

The study showed that there were association of PMTCT service utilization with women education, having monthly income ≥1000 ETB, residence around PMTCT service site than those who cannot read and write, have monthly income <1000 ETB, and live far from PMTCT sites, respectively. Moreover, the association was significant with the *P* values 0.001, 0.001, and 0.001, respectively (Table 2).

Different factors which prevent pregnant women from using PMTCT service utilization were identified by pregnant women attending ANC. These include excess waiting time in uncomfortable hot waiting rooms, limited physical access to health facilities, and lack of transportation system. But, the association was not significant with the *P* values of 0.32, 0.22, and 0.44, respectively.

**Figure 3:**
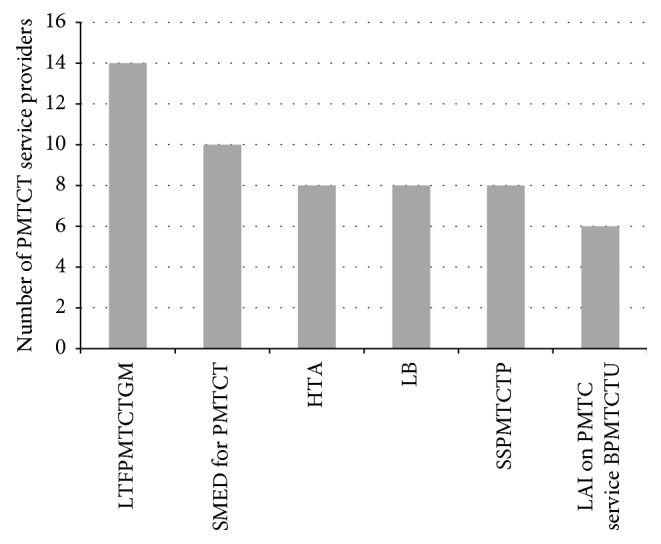
Factors listed by health professionals as a major challenge in utilization of PMTCT service in five health facilities studied from December 2014 to April 2015 in the Afar regional state, Ethiopia. LTFPMTCTGM: lack of training following PMTCT guideline modification; SMED for PMTCT: shortage of materials, equipment, and drug for PMTCT, HAT: hot temperature of the area; LB: language barrier; SSPMTCTP shortage of skilled PMTCT providers; LAI on PMTC service BPMTCTU: lack of awareness and ignorance on PMTCT service by PMTCT users.

Moreover, PMTCT providers were also asked on the list of 10 potential barriers identified from literatures either the barriers listed are a challenge or not in their health facility in utilization of PMTCT service. PMTCT providers were asked to respond by saying as not a challenge, simple challenge, or major challenge. Among the potential factors listed, educational level and time shortage of PMTCT service users were not considered as a major challenge by all PMTCT providers in their health facility. But lack of up-to-date training following PMTCT guideline modification was considered as a major challenge by 14 (63.6%) service providers (Figure 3).

## 4. Discussion

The World Health Organization identified 22 priority countries with the highest PMTCT service need. Among these, the top 10 countries including Ethiopia account 75% from the total global PMTCT service need. It was estimated that the effective scaling up of PMTCT interventions in these countries would prevent over 250,000 new infections annually [12].

The knowledge of pregnant women included in the present study about MTCT of HIV was quite higher (46.4%) than the Ethiopian Demographic and Health Survey (EDHS) data of the region in 2014 (36%). This variation might be explained that the present study was conducted in urban and semiurban community compare to the Ethiopian Demographic and Health Survey (EDHS) data conducted in the total Afar population. This result, however, was lower than a similar study conducted in Addis Ababa, 90.3% [8], and Sebeta town, Ethiopia (64.9%) [13]. This variation might be due to the difference in education level where 35.7% of the study participants in the present study were illiterate which is higher than those in the study in Addis Ababa (21%) [8] and Sebeta town (17.1%) [13]. Moreover, there was a difference in urbanization between the country capital Addis Ababa and agropastoral community of the present study area.

From the total pregnant women studied, 246 (70.9%) were tested for HIV. This was lower than a study conducted in Addis Ababa [8], Jinka towns [14], Sebeta town [13], and Horo Guduru Wollega zone [10] of Ethiopia where 94%, 96.4%, 86.9%, and 83.8% got tested for HIV, respectively. This might be due to the living style (agropastoral) and educational status difference among the population studied as well as study time gap. The present study also showed that 28% of pregnant women tested for HIV were by their husband/partner support. Moreover, the prevalence of HIV in the present study was 13.8%. This was higher than from EDHS data on HIV prevalence in Afar region (9%) [11] and studies conducted in Jinka town [14] and Horo Guduru Wollega zone [10] which were 3.4% and 1.3%, respectively. This can be explained that the present study area is located in the main import-export road corridor of Ethiopia which runs from Addis Ababa to Djibouti hosting many track drivers.

Though PMTCT service is known to reduce the transmission of HIV from mother to child, its use has been limited because of various barriers. The present study had revealed different barriers that might hinder the success of the PMTCT program in both health centers and hospitals. The barriers identified in the present study by pregnant women as a challenge to use PMTCT service were lack of awareness and information access, being uneducated, physical inaccessibility of PMTCT service sites, low income, low involvement of male partners, fear of discrimination and stigma by the community, and financial and transportation problems. This finding agreed with a study in Addis Ababa (Ethiopia) which identified inconsistent supplies, inadequate counseling rooms, limited access to PMTCT service information, HIV/AIDS-related stigma and discrimination in the community, low male involvement, inadequate knowledge, inaccessibility of PMTCT services (unpublished MSC thesis). Moreover, the barriers identified in the present study finding also aggress with a study done in the Horo Guduru Wollega zone of Ethiopia which reported residence, mother's educational status, and male partner involvement during ANC [10].

Moreover, PMTCT service providers in the present study identified lack of trainings following PMTCT guideline modification, hot temperature of the area, shortage of skilled PMTCT provider, lack of awareness of PMTCT service users, and lack of materials and equipment as a major challenge to use PMTCT service. Similar reports on some factors were also identified in the study done in Cameroon as a challenge in using the service which includes low level of education and level of male involvement [15]. Furthermore, the present study goes in line with the study conducted in Addis Ababa, Ethiopia, which reported shortage of PMTCT-trained staff, physical inaccessibility of health facilities, inadequate maternal health services, lack of separate and adequate space for PMTCT services, and economic factors as a major barrier for using PMTCT service [8].

## 5. Limitation

Since the study is conducted on those pregnant mothers attending their ANC follow up in the health facility, the study result cannot reflect the situation in the whole Afar regional state since there are mothers who do not seek ANC follow up. The sampling technique used in the present study was convenient sampling technique which will not give equal chance to be included in the study for all pregnant women using the health facility. Since 35.7% of our study participants were illiterate, there may be a recall bias which will intern affect the result the study. Furthermore, the present study is a cross-sectional study, and causality cannot be established between the independent and dependent variables.

## 6. Conclusion

Overall, the present study showed promising findings on knowledge of mothers on MTCT of HIV and level of PMTCT service utilization. On the contrary, there were also different major barriers identified in PMTCT service utilization by pregnant women and PMTCT service providers working ANC. These major barriers identified by pregnant women in using PMTCT service include being uneducated, physical inaccessibility, and limited knowledge of PMTCT service sites. Moreover, lack of up-to-date PMTCT training, lack of materials and equipment for PMTCT service, hot temperature of the area, language barrier, and low number of PMTCT providers were the major barriers identified by the PMTCT service provider in providing PMTCT service.

Thus, to improve the accessibility and utilization of PMTCT service, major investment is required by the regional and federal government of Ethiopia to minimize major barriers identified by both service users and providers. These can be done thorough expansion of formal education, construction of roads leading to PMTCT sites, expansion of PMTCT sites, and enhancing the overall economic status of the community. On the contrary, we recommend the local and zonal health office to perform activities which require low financial investment like creating awareness on the significance of PMTCT service in the community and role of husband in PMTCT service through health education, providing up-to-date trainings for PMTCT service providers, ensuring constant supply of materials and reagents for PMTCT service.

Moreover, heath education by health extension workers (HEWs) must be given for pregnant women and to the community at large so as to reduce the HIV positivity in pregnant women identified in the study. Moreover, we also recommended HEWs to provide health education for pregnant women to deliver in health facilities than at home with traditional birth attendant (TBA) involvement. Finally, we recommend similar study to be conducted covering the large area of agropostural community of the Afar regional state of Ethiopia to get a better picture.

## Figures and Tables

**Table 1:** Sociodemographic characteristics of the pregnant women attending ANC service in selected health facilities of Afar region, Ethiopia, from December 2014 to April 2015.

Sociodemographic variables	Categories	Frequency	Percent
Age group (*n* = 347)	<18 years old	49	14.1
	19–25 years old	121	34.9
	26–30 years old	100	28.8
	31–35 years old	60	17.3
	36–40 years old	9	2.6
	>40 years old	8	2.3

Marital status (*n* = 347)	Unmarried	9	2.6
	Married	310	89.3
	Divorced	28	8.1

Educational status (*n* = 347)	Cannot read and write	124	35.7
	1–4 (1st cycle)	97	28.0
	5–8 (2nd cycle)	97	28.0
	High school and above	29	8.4

Residence (*n* = 347)	Urban	253	72.9
	Rural	94	27.1

Religion (*n* = 347)	Orthodox	68	19.6
	Muslim	268	77.2
	Others	11	3.2

Ethnicity (*n* = 347)	Afar	254	73.20
	Amhara	42	12.10
	Tigre	35	10.08
	Oromo	10	10.08
	Others	6	1.72

Address (*n* = 347)	Dubti hospital	129	37.2
	Aysaita District Hospital	69	19.9
	Logia health center	66	19.0
	Samara Health center	47	13.5
	Chifra Health Center	36	10.4

Monthly income in birr (*n* = 347)	<500 birr	75	21.6
	500–1000 birr	113	32.6
	1000–1500 birr	53	15.3
	>1500 birr	54	15.6
	I do not know	52	15.0

**Table 2:** Association of sociodemographic and other possible potential risk factors with PMTCT service utilization in relation to ANC service in selected health facilities of Afar region, Ethiopia, from December 2014 to April 2015.

Variables	Categories	PMTCT utilization related to ANC service		COR (95% CI); *P* value	AOR (95% CI); *P* value
		Utilized	Not utilized		
Residence near PMTCT service site	Yes (≤1 km)	244 (70.3%)	28 (8.07%)	2.5 (1.25–3.9); 0.001	6.5 (2.35–18.40); 0.001
	No	106 (30.54%)	25 (7.20%)	1	1

Educational status	Could read and write	208 (59.94%)	15 (4.32%)	3.4 (2.3–7.46); 0.001	11.3 (2.8–15); 0.001
	Could not read and write	97 (27.95%)	27 (7.78%)	1	1

Knowledge about PMTCT service	Yes	177 (51%)	13 (3.74%)	3.09 (1.54–6.2); 0.001	0.9 (0.8–5.25); 0.08
	No	128 (39.90%)	29 (8.35%)	1	1

Income level per month	≥1000 ETB	139 (40.05%)	20 (5.76%)	3.4 (1.56–8.04); 0.001	5.6 (2.05–15.03); 0.001
	<1000 ETB	166 (47.53%)	22 (6.32%)	1	1

Access for the PMTCT service site	Yes (≤1 km)	244 (70.3%)	28 (8.07%)	2.5 (1.25–3.9); 0.001	6.5 (2.35–18.40); 0.001
	No (>1 km)	61 (17.58%)	14 (4.03%)	—	1

AOR = adjusted odds ratio; COR = crude odds ratio; 1 = reference value; PMTCT = prevention of mother-to-child transmission.

## Data Availability

The data used to support the findings of this study are available from the corresponding author upon request.

## Abbreviation

AIDS: Acquired immune deficiency syndrome
ANC: Antenatal care
ART: Antiretroviral therapy
EDHS: Ethiopian Demographic and Health Survey
ETB: Ethiopian birr
HEW: Health extension worker
HIV: Human immuno-deficiency virus
MOH: Ministry of Health
MTCT: Mother-to-child transmission
NIH: National Institutes of Health
PMTCT: Prevention of mother-to-child transmission
VCT: Voluntary counseling and test
WHO: World Health Organization.
